# Current Practice in Obstetric Anesthesia and Analgesia in Public Hospitals of Greece: A 2016 National Survey

**DOI:** 10.4274/balkanmedj.2018.0083

**Published:** 2018-09-21

**Authors:** Chryssoula Staikou, Alexandros Μakris, Kassiani Theodoraki, Athanasia Τsaroucha, Amalia Douma, Eleni Μoka, Eleni Αrnaoutoglou, Tilemahos Paraskevopoulos, Ioanna Siafaka, Efi Stavropoulou, Eriphili Αrgyra

**Affiliations:** 1 Clinic of Anesthesia, Aretaieio Hospital, National and Kapodistrian University of Athens School of Medicine, Athens, Greece; 2 Clinic of Anesthesia, Asklipieio Voulas General Hospital, Athens, Greece; 3 Clinic of Anesthesia, Georgios Gennimatas General Hospital, Athens, Greece; 4 Clinic of Anesthesia, Creta Interclinic Hospital, Heraklion, Crete, Greece; 5 Clinic of Anesthesia, General University Hospital of Larissa, Larissa, Greece; 6 Clinic of Anesthesia, General Hospital of Attica KAT, Athens, Greece

**Keywords:** Analgesia, healthcare, obstetrical, regional anesthesia, survey

## Abstract

**Aims:**

This descriptive survey was to evaluate the use of regional anesthesia in obstetrics in Greek public hospitals.

**Methods:**

The survey was conducted between March and August 2016. A structured questionnaire was sent to 50 anesthesia departments in Greek public hospitals with obstetric units.

**Results:**

The response rate was 94%. Data corresponding to 9475 cesarean and 8155 vaginal deliveries were collected. Regional anesthesia was used in 69.2% of all cesareans, with single shot spinal being the most popular (44.3% of all cesareans). Combined spinal-epidural anesthesia was used in 18.1% of all cesareans (35.1% in hospitals of Athens versus 7.9% outside Athens, p<0.001). Post-cesarean analgesia was applied with simple analgesics and systematic opioids (78.6%). Long-acting spinal opioids were rarely used (4.4% of spinal and spinal/epidurals). Labor epidural analgesia was applied in 19.1% of all vaginal deliveries (30.3% in Athens versus 13.1% outside Athens, p<0.001). Paracetamol and pethidine represented the standard labor analgesics in 48.9% and 55.3% of all hospitals. Intravenous remifentanil was used in 10.6% of hospitals (50% in Athens versus 2.5% outside Athens, p=0.002). In 48.9% of hospitals, mainly outside Athens, the anesthesiologists did not get involved in labor analgesia.

**Conclusion:**

Regional anesthesia is the most common practice for cesareans in Greek public hospitals; however, the percentage of general anesthesia remains high. In addition, the use of labor epidural analgesia is limited in hospitals outside Athens.

Central neuraxial blocks are commonly practiced regional anesthesia (RA) techniques in the perioperative period for obstetric anesthesia and analgesia that are associated with both maternal and neonatal safety (1). RA is the standard method for the majority of uncomplicated cesarean deliveries (CDs). Moreover, RA is gaining popularity in selected complicated cases where general anesthesia (GA) was traditionally preferred, such as in hypertension/preeclampsia or even in abnormal placentation with anticipated hemorrhage (1,2). Data regarding the use of RA in the obstetric population of European and other countries are limited (3,4,5,6). Therefore, such studies can help in the improvement of peripartum anesthesia care because the data can be used for inter- and intra-national comparisons that reveal the current trends, the progress or drawbacks made over the years, and problems that should be addressed (7).

The present survey was conducted in 2016 and its primary aim was to evaluate the application of regional techniques, in terms of frequency and popularity, for obstetric anesthesia/analgesia in Greek public hospitals and to identify any factors that may influence their use in daily clinical practice. Therefore, data regarding familiarity, education and training in regional techniques, and human and technical resources in these public hospitals were also recorded and assessed.

## MATERIALS AND METHODS

A nationwide descriptive survey was conducted between March 1^st^ and August 31^st^, 2016. The survey was approved by the Institutional Review Board (approval number: B-106/04-30-2015) and was endorsed by the Greek Section of the European Society of RA and Pain Therapy (ESRA-Hellas). The authors designed a five-part questionnaire on the use of RA in obstetric clinical practice in Greek public hospitals (Appendix 1). The questionnaire had twenty-five questions with two types of answer formats (i.e., filling in the blank with numbers and selecting the appropriate boxes). The format, structure, and content of the questionnaire were assessed by five Senior Anesthesiologists experienced in regional and obstetric anesthesia. The final questionnaire was reviewed and approved by the ESRA-Hellas Scientific Committee. Potential respondents were the directors of anesthesia departments of all Greek public hospitals with obstetric units. We identified 65 public hospitals with registered obstetric units and we contacted all of them. However, 15 out of the 65 hospitals contacted reported that their obstetric units are currently inactive, or do not function regularly due to lack of personnel. In January 2016 the questionnaires were sent by email to the remaining 50 anesthesia departments with active obstetric units. In the email, an accompanying letter explained the survey objectives and invited anesthesia departments directors to participate and complete the questionnaire, anonymously if they preferred. In February, a reminder email was sent, and in case of no response, contact was attempted by fax, and/or telephone. All participants were informed that data would be collected prospectively for six months, starting on March 1^st^ and ending on August 31^st^, 2016. All the participants were instructed to be extremely careful and accurate with data acquisition and reporting. They were also informed that participation in the survey was voluntary and without monetary compensation.

### Statistical analysis

The returned questionnaires were evaluated for data completion and missing values and were analyzed with the SigmaPlot for Windows v.11.0 statistical software (Systat Software, Inc., San Jose, CA, USA). Descriptive statistics (frequency distributions) were used to summarize the data; therefore, the results are presented as total numbers, frequencies, or percentages. Comparisons between the characteristics and practices in hospitals of Athens and hospitals in other cities were assessed with the chi-square test or Fisher’s exact test. Results were considered statistically significant if p<0.05.

## RESULTS

Forty-seven (47/50) completed questionnaires were returned and analyzed (response rate of 94%). The acquired data corresponded to 17630 deliveries (9475 cesarean and 8155 vaginal) performed during the 6-months study period in public hospitals in all the Greek geographical regions. RA was used in 6562 (69.2%) out of 9475 CD cases, with single shot spinal block being the most popular technique preferred in 44.3% of the RA cases. Combined spinal-epidural anesthesia (CSEA) was used in 18.1% and epidural anesthesia in 6.7% of the CDs cases. GA was used in 2913 (30.7%) of CD cases. The main reason was parturient refusal for RA or request for GA (41.3% of GAs), while emergency CD or not enough time for RA accounted for 20.4%, parturient pathology accounted for 7.2% and other reasons for 30.9% of the total number of GAs. Analgesia after CD was based on simple analgesics and systemic opioids (78.6% of the CDs). When an epidural or a CSEA was performed, the epidural catheter was maintained for postoperative analgesia in 40.7% of the cases. Spinal opioids with long duration of action were used only in 4.4% of spinal and CSEA cases. Labor epidural analgesia was used in 1562 out of 8155 (19.1%) vaginal deliveries (VDs). Among other analgesic methods, paracetamol and intramuscular pethidine, were the most popular, used in 48.9% and 55.3% of the responding hospitals. Parturient-controlled analgesia with intravenous remifentanil was used in 10.6% and blocks performed by the obstetrician were reported in 8.5% of the responding hospitals. In 29.7% of the hospitals, no analgesia was offered to the parturients, while in 48.9% of the hospitals, the anesthesiologists were not involved in the delivery suite. Regarding equipment availability, all the responding hospitals (100%) reported to have adequate supplies of spinal needles and epidural sets. In 85.1% of the hospitals, CSEA sets were also available. Ultrasound machines were available in 23.4% of the anesthesia departments; however, only 8.5% reported its use in obstetric cases. Complications related to RA were reported by 27.6% of the hospitals. Accidental dural puncture resulting in headaches occurred in 53 out of 3924 women (1.35%) who underwent epidural or CSEA.

**Figure 1:**
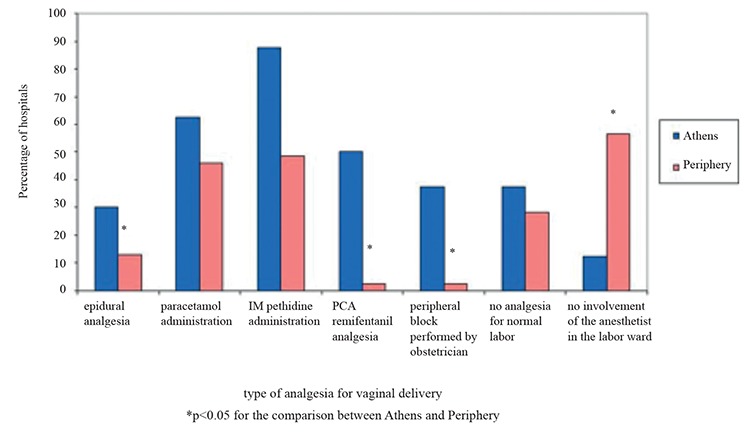
Type of analgesia provided for vaginal delivery in hospitals of Athens and other cities.* PCA: patient-controlled analgesia*

In the responding anesthesia departments, the majority of consultant anesthesiologists (96.3%) were involved in obstetric cases. Of them, 74.9% were familiar with the single-shot-spinal block technique and 70.4% were familiar with the epidural and CSE technique. Nevertheless, 14.7% of the anesthesiologists reported that they do not perform RA and provide only GA in obstetric cases. Some of the reasons for not applying RA were parturients’ refusal for RA (68%), anesthesiologists’ preference (36.1%), obstetricians’ preference (31.9%), and lack of training/education in 17% of the hospitals surveyed. According to the analyzed data, 3546 CDs and 2842 VDs were performed in hospitals of Athens and 5929 CDs and 5313 VDs were performed in other cities. Comparisons between Athens and other cities hospitals showed that RA, especially CSEA, was used more frequently in hospitals of Athens, while single-shot-spinal block was more popular in hospitals outside Athens (p<0.001) (Table 1). Also, labor epidural analgesia and intravenous remifentanil were used much more often in hospitals of Athens (Figure 1). A relatively high percentage of anesthesiologists were not involved in the delivery suite; this attitude was mainly observed in hospitals outside Athens (56.4%) than in hospitals of Athens (12.5%), p=0.048 (Figure 1). In 62.5% of Athens, there was an ultrasound machine available for use versus 15.3% in hospitals outside Athens (p=0.011). Finally, post-dural puncture headache was more frequent in hospitals outside Athens (36/1607, 2.2% of epidurals/CSEA) than in hospitals of Athens (17/2317, 0.7%), p<0.001.

## DISCUSSION

According to the results of the present survey, RA was used in the majority of CDs in Greek public hospitals (about 70%). The single-shot-spinal block was the most popular technique, especially in hospitals outside Athens. The rate of GA for CDs was significantly lower (30.7%) than RA, but still quite high, especially when compared with the rates reported by other countries (6,8,9,10). In a recent analysis of a USA registry, the overall rate of GA usage nationally for CDs is 5.8% (8), while similar low rates were reported in Germany (less than 10%) (9), in Switzerland (5%) (10), and in Taiwan (3.9%) (6). Nevertheless, there are recent surveys reporting even higher rates of GA than ours; in the Czech Republic, almost half of the CDs (47%) are performed under GA (5). In Western countries, GA is almost exclusively used in emergency CDs or in case of RA failure (8,9,10). An interesting finding of the survey was that the main reason for choosing GA over RA was parturient preferences (over 40% of GAs). It is possible that the parturients in Greece, especially those living in rural areas, immigrants, and refugees, are not well informed about the advantages of RA and this may be an area for discussion and future improvement. It could also indicate that anesthesiologists prefer to follow parturients’ preferences for GA and do not try to persuade them to receive RA, especially when language or religion barriers exist. RA for CD, especially CSEA, is mostly used in hospitals of Athens, probably because it requires more expertise, and represents a relatively novel addition to the anesthesiologists’ armamentarium in obstetric cases compared to GA or spinal techniques. Since most teaching and university hospitals are in hospitals of Athens, any new techniques are integrated into daily practice faster. When RA is used, anesthesiologists in the cities outside Athens prefer the single-spinal block anesthesia method since it is simpler and costs less than CSEA. Regarding labor analgesia, the placement of the epidural catheter was performed in less than 1/5 vof VDs. Similar figures have been reported in the last decade in Germany (17.5%±12.6%) and Norway (25.9%) (11,12). The rates have increased in UK (over 33%) and are significantly higher in Belgium (68%) and USA (77%) (13,14). This low percentage in Greece can be explained by the fact that many anesthesiologists do not get involved in the delivery suite. This attitude is mainly observed in hospitals outside Athens and is possibly explained by the lack of personnel, which has been further exaggerated by the financial crisis. Epidurals and parturient-controlled analgesia with intravenous remifentanil are also used more frequently in hospitals of Athens than in other cities. A possible explanation is a better-organized delivery suite and more specialized obstetric anesthesiologists, personnel, and services in hospitals of Athens. The reported incidence of accidental post-dural puncture headache was 1.35%, which does not differ from that described in the literature. According to published data, inadvertent dural puncture voccurs in 1.5% of epidurals, and up to 88% of dural punctures in obstetric cases result in the development of severe headache (15,16,17). Therefore, we can conclude that epidurals and CSEA, when used in Greek public hospitals, are performed by skilled anesthesiologists. The present survey has certain limitations. This is the first national Greek survey on obstetric anesthesia/analgesia; therefore, there are no previous data to compare our findings or conclude if any progress and improvement has been made. Also, the questionnaire did not investigate parturients’ satisfaction regarding the anesthetic/analgesic methods and service provided. On the other hand, the main goal of the survey, the evaluation of current nationwide usage of obstetric RA in public hospitals was achieved since the response rate from several Greek hospitals was very high (94%). For most surveys, the goal of researchers is a 60% response rate, while an 80% response rate may be considered representative (18). In similar obstetric anesthesia surveys from other European countries published in the last 6 years, the response rate was quite lower than ours, ranging between 50% and 68% (4,5,19,20). Another advantage of the study is its prospective design; the collaborators from public hospitals were asked to follow and record each consecutive obstetric case from the first until the last day of the study period, thus reducing bias and restrictions associated with retrospective data collection.

Under the present survey design, we found that RA represents the most common practice for CDs in Greek public hospitals; however, the percentage remains low compared to other European countries. Also, the use of epidural labor analgesia is quite limited and in many hospitals, especially outside Athens, the anesthesiologists are not involved in labor analgesia and peripartum care of women undergoing VD.

## Figures and Tables

**Table 1:**
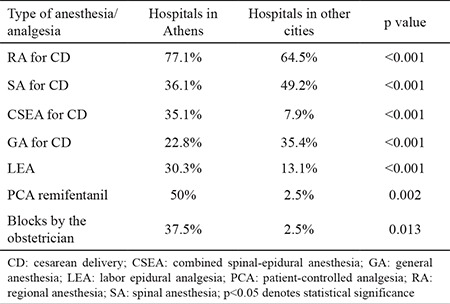
Comparisons between hospitals in hospitals of Athens and other cities regarding the anesthetic/analgesic practice in obstetrics
